# Monocyte‐crosstalk drives interferon‐mediated signaling following SARS‐CoV‐2 exposure

**DOI:** 10.15252/msb.202211256

**Published:** 2022-09-12

**Authors:** Sebastian J Theobald, Jan Rybniker

**Affiliations:** ^1^ Department I of Internal Medicine, Faculty of Medicine University of Cologne Cologne Germany; ^2^ Center for Molecular Medicine Cologne (CMMC), Faculty of Medicine University of Cologne Cologne Germany; ^3^ German Center for Infection Research (DZIF) Partner Site Bonn‐Cologne Cologne Germany

**Keywords:** Immunology, Microbiology, Virology & Host Pathogen Interaction

## Abstract

Cells of the innate immune system represent the first line of defense against SARS‐CoV‐2 and play an essential role in activating adaptive immunity, which mediates long‐term protection. In addition, the same cells are key drivers of tissue damage by causing the hyperinflammatory state and cytokine storm that makes COVID‐19 a deadly disease. Thus, careful dissection of the host–pathogen interaction on a cellular level is essential to understanding SARS‐CoV‐2 pathogenesis and developing new treatment modalities against COVID‐19. In their recent work, Goffinet and colleagues (Kazmierski *et al*, 2022) investigate the cell‐intrinsic responses of human primary peripheral blood mononuclear cells (PBMCs) exposed to SARS coronaviruses.

## Graphical abstract

It remains unknown whether innate immune signaling events require infection and active production of SARS‐CoV‐2 in immune cells. Goffinet and colleagues (Kazmierski *et al*, 2022) address this question by studying cell‐intrinsic responses of human PBMC exposed to SARS coronaviruses.

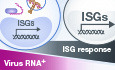

The authors first use a single‐cell RNA‐sequencing approach on all mononuclear cells and then focus on monocytes, which represent classical cells of the innate immune system. They show that SARS‐CoV‐2‐exposed cells mount a JAK/STAT‐dependent innate immune response, which seems to be mediated primarily by interferons (IFN). In SARS‐CoV‐2‐infected patients, type I IFNs and interferon‐stimulated genes (ISG) are known to orchestrate an efficient adaptive immune response against the virus. In light of the importance of IFN signaling, the here described *ex vivo* approach exploiting human PBMCs exposed to SARS‐CoV‐2 particles represents a highly valuable model to study SARS‐CoV‐2 host–pathogen interactions and to decipher cell‐specific production of cytokines (Fig 1).

**Figure 1:**
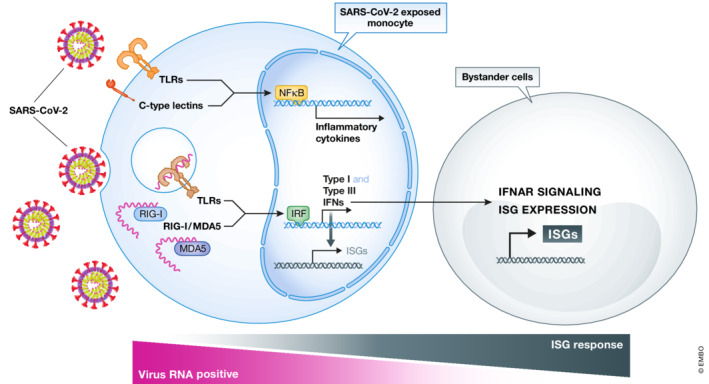
Molecular pathways and crosstalk in SARS‐CoV‐2‐infected PBMCs Monocytes react via different molecular pathways towards a SARS‐CoV‐2 infection. Surface molecules, such as C‐type lectins and Toll‐like receptors, are able to detect molecular patterns on SARS‐CoV‐2, which as a consequence leads to NfĸB activation and production of proinflammatory cytokines. On the contrary, SARS‐CoV‐2 single‐stranded RNA triggers intracellular signaling cascades, such as Toll‐like receptors or RIG‐I/MDA5. Recognition of SARS‐CoV‐2 by monocytes leads to the activation of interferon‐response genes and consequently to the section of type I and III interferons. Bystander monocytes, which are virus RNA‐negative, are sensitized and therefore activate interferon‐stimulated gene (ISG) signatures to higher levels than RNA‐positive cells.

SARS‐CoV‐2‐specific T‐ and B‐cell responses have been broadly studied over the course of the pandemic. However, considerably less attention has been paid to innate immune triggers and signaling. Nevertheless, almost 3 years after the start of the pandemic, some features of innate immune signaling induced by SARS coronaviruses have been elucidated. SARS‐CoV‐2 can trigger several different innate immune pathways (Diamond & Kanneganti, 2022; Paludan & Mogensen, 2022). For instance, recent studies show that innate immune recognition of viral RNA via RIG‐1/MDA5 and TLR7/8 activates interferon‐signaling cascades leading to the secretion of type I and III IFNs (Schultze & Aschenbrenner, 2021; Thorne *et al*, 2021; Diamond & Kanneganti, 2022) (Fig 1). This discovery correlates well with findings made in the systemic evaluation of SARS‐CoV‐2‐exposed immune cells discussed here.

A key question is whether these events require infection and active production of SARS‐CoV‐2 particles in cells of the innate immune system. Goffinet and colleagues address this topic carefully by showing that, in the cell culture of SARS‐CoV‐2‐exposed PBMCs, viral RNA remains associated with cells for up to several days; however, several lines of evidence indicate that human blood‐derived immune cells fail to support productive viral infection. This finding has also been confirmed by other studies independently (Yang *et al*, 2020). Furthermore, virus–receptor interaction required for the activation of innate immune cell signaling does not require the common SARS‐CoV‐2 receptor, the angiotensin‐converting enzyme 2 (ACE2). While receptor requirements have not been addressed in this work, there is evidence that the major antigen of SARS coronaviruses, the spike protein, functions as a pathogen‐associate molecular pattern on innate immune cells and triggers C‐type lectin and TLR‐2‐dependent signaling cascades, which in turn leads to the activation of inflammasomes and the secretion of proinflammatory cytokines, such as IL‐1, IL‐6, and TNF‐α (Theobald *et al*, 2021; Sefik *et al*, 2022). However, at least for activation of the NLRP3 inflammasome and IL‐1 secretion, peripheral blood monocytes require a certain degree of prestimulation (e.g., via active infection or vaccination of the human host) (Rodrigues *et al*, 2021; Theobald *et al*, 2022). This may explain the lack of expression of proinflammatory cytokines, including IL‐6, TNF‐α, and IL‐1, observed in the study of Goffinet and colleagues, which uses PBMCs of healthy individuals for the *ex vivo* SARS‐CoV‐2 exposure experiments. Therefore, it seems like the described model rather mimics a situation in which protective IFN‐driven immunity is induced as we would expect in mild forms of the disease. Surprisingly though, single‐cell RNA‐seq experiments reveal that the SARS‐CoV‐2 RNA‐negative bystander monocytes induce the JAK/STAT‐dependent ISG expression signature to a greater extent than viral RNA‐positive cells (Fig 1). Goffinet and colleagues speculate that SARS coronavirus‐encoded IFN antagonists dampen the innate immune response in SARS‐CoV‐2 RNA‐positive cells, whereas RNA‐negative bystander monocytes seem to receive signals that activate the protective IFN‐dependent signature required to mount a robust antiviral immune response. Understanding the exact mechanism behind this virus‐induced dampening effect represents a formidable task with great translational value. Goffinet and colleagues now have provided the cornerstone for these important investigations.
